# Iron Deficiency-Induced Changes in the Hippocampus, Corpus Striatum, and Monoamines Levels That Lead to Anxiety, Depression, Sleep Disorders, and Psychotic Disorders

**DOI:** 10.7759/cureus.18138

**Published:** 2021-09-20

**Authors:** Hira E Shah, Nitin Bhawnani, Aarthi Ethirajulu, Almothana Alkasabera, Chike B Onyali, Comfort Anim-Koranteng, Jihan A Mostafa

**Affiliations:** 1 Medicine, California Institute of Behavioral Neurosciences & Psychology, Fairfield, USA; 2 Internal Medicine, California Institute of Behavioral Neurosciences & Psychology, Fairfield, USA; 3 General Medicine, California Institute of Behavioral Neurosciences & Psychology, Fairfield, USA; 4 Psychiatry, California Institute of Behavioral Neurosciences & Psychology, Fairfield, USA

**Keywords:** iron deficiency anemia (ida), depression, anxiety, hippocampal tissue, corpus striatum, sleep disorders, psycho-behavioral

## Abstract

Iron deficiency anemia caused by severe iron deficiency in infancy is associated with poor health and severe neurological impairment such as mental, motor, social, emotional, neurophysiological, and neurocognitive dysfunction. The behavioral effects of iron deficiency can present themselves in infancy, but they are also found in adulthood. Some behaviors can start in childhood but persist throughout adulthood.

The behaviors that are particularly often seen in infants and children include wariness and hesitance, lack of positive affect, and diminished social engagement. The affected behaviors in adults include anxiety, depression, higher complex cogitative tasks, and other psychological disorders.

The mechanisms of how iron deficiency affects behavior include affecting the hippocampus, the corpus striatum, and certain neurotransmitters. The hippocampus is a brain region that is essential for memory, learning, and other purposes. The hippocampus is very sensitive to lack of Iron during early development. The corpus striatum dispatches dopamine-rich projects to the prefrontal cortex, and it is involved in controlling executive activities such as planning, inhibitory control, sustained attention, working memory, regulation of emotion, memory storage and retrieval, motivation, and reward. Iron deficiency has been known to cause changes in behavioral and developmental aspects by affecting neurotransmitters such as serotonin, noradrenaline, and dopamine.

Iron deficiency causes behavior changes that can present in infancy and, even if corrected postnatally, it can have long-lasting effects well into adulthood.

## Introduction and background

"The goal of early childhood education should be to activate the child's own natural desire to learn" [1]. This is a quote from Maria Montessori, an Italian physician known for her educational methodology that builds on the way children learn naturally. She started the first Montessori school, the Casa Dei Bambini, or Children's House in Rome, on January 6, 1907. Unfortunately, the ability of children to excel at learning is often hindered by iron deficiency. It has been postulated that iron deficiency contributes to deficient initial reading skills [DIRS]. While other causes can affect learning disabilities, it should be considered one of the components involved in learning deficit. With this in mind, investigations should be carried out to analyze and reduce the consequences of iron deficiency [2].

Moreover, another fundamental reason to investigate iron deficiency is that, as claimed by the WHO, iron deficiency is the leading nutritional disorder globally, and it predominately affects women and preschool children [3]. Before further exploration of iron deficiency, it is important to learn about Iron. Iron, an essential nutritional component for all life forms, is necessary for many vital functions, including oxygen transport, cell respiration, immunity, neurotransmitter function, and DNA synthesis [4,5]. 

Iron deficiency anemia caused by severe iron deficiency in infancy is associated with poor health and severe neurological impairment such as mental, motor, social, emotional, neurophysiological, and neurocognitive dysfunction [6]. According to Kim et al., iron deficiency affects the neurotransmitter glutamate and γ-aminobutyric acid (GABA) hemostasis, which causes deficits in learning, memory, learning, and behavior [7]. Additionally, it also contributes to emotional and psychological problems.

This article will focus on the behavioral effects of iron deficiency, which can present itself in infancy but are also found in adulthood. Some behaviors can start in childhood but persist throughout adulthood. One such typical behavior found in childhood and adulthood pagophagia, ice craving, is often iron deficiency [8]. Some of the behaviors presenting in infants with chronic severe iron deficiency include increased fearfulness, unhappiness, fatigue, low activity, wariness, solemnity, and proximity to the mother during free play [9-15]. In adulthood, the behavioral disorders linked to iron deficiency are depression, anxiety disorders, sleep disorders, and psychotic disorders [16].

In this study, we plan to explore the effects of iron deficiency on behavior and, in some instances, behavior on iron deficiency. Moreover, in our exploration, we will delve into some of the mechanisms of how behavior is affected. 

## Review

What behaviors are affected by iron deficiency

When we discuss what type of behaviors iron deficiency affects, we first need to split the behavioral effects into two categories: behaviors that affect children and behaviors that affect adults.

Iron Deficiency-Induced Wariness, Hesitance, Social Disengagement in Children

In children, it has been postulated that iron deficiency can affect neuropsychological and social-emotional functions as well as cause anti-social behavior [17]. The timing of the iron deficiency is also important. If iron deficiency occurs in early life, it will result in a developmental delay [18]. Studies have also shown that improved dietary iron supplementation was not effective if the iron deficiency transpired in early life and over a long time [19]. The behaviors that are often seen in infants and children include wariness and hesitance, lack of positive affect, and diminished social engagement [20]. This wariness and hesitance were shown in several studies that have been performed on rat infants with iron deficiency. Lozoff et al. noticed those anemic infants that were afflicted with iron deficiency displayed "increased fearfulness" even after iron therapy was initiated [21]. Deinard et al. displayed that infants with slight iron deficiency were prone to pronounced fearfulness [22]. Beard et al. also studied related behaviors in a study of iron-deficient rats at six weeks of age [23]. They noticed anxiety-driven activities with light/dark box measurements of distance traveled, repeated movements, number of nose-pokes, center time, and habituation to a novel environment. These studies say that iron deficiency in infants can lead to behavioral tendencies that affect confidence, social interactions, and anxiety. If the deficiency occurs in very early life and/or for a prolonged time, the effects are not reversible with iron supplementation. So, while these studies provided significant insights into how behaviors are affected by iron deficiency, it is essential to remember that a good number of studies performed were on animals.

Iron Deficiency-Induced Depression, Negative Symptoms of Psychosis, and Complex Cognitive Task Dysfunction in Adults

Now we will pivot and look at the behavioral effects of iron deficiency in adults. One such behavior that has been shown by several authors is depression. Steward and Hirani studied about 2,000 individuals over age 65, and they surmised that lower than normal levels of hemoglobin, ferritin, and transferrin were linked with depressive symptoms [24]. Other studies linked depression and iron management guidelines in diverse groups, including female medical students, adult workers [25-27], and even in animal studies [28,29]. Additionally, consistent results were seen in patients with maternal iron deficiency anemia, which can affect postpartum reasoning, emotional reactions, and postpartum depression [30,31].

Another behavior that was shown to be affected by iron deficiency was performing complex cognitive tasks. A double-blind, randomized, interventional study performed by Wenger et al. sought to show the behavioral changes in Rwandan female university students [32]. They selected 55 women between the ages of 18-27 years with low iron levels (serum ferritin < 20 µg/L). They were then randomly designated to either ingest iron-fortified beans (86.1 parts per million iron) or comparison beans (50.1 parts per million iron) daily for 18 weeks. The iron level was gauged by ferritin, hemoglobin, transferrin receptor, and body iron. The subjects' cognitive performance was measured with five computerized tasks with concurrent electroencephalography performed at baseline and end line. The study concluded that the EEG and behavioral results indicated that changes in iron level were most likely to affect complex cognitive tasks such as instant-by-instant control of attention and word-by-word retrievals from memory in creating a spoken sentence. So, this study is suggesting that low iron status contributes to slower response times and difficulty with cognitive processing at mid to high levels.

One additional study found that iron deficiency contributed to the negative effects of schizophrenia. Kim et al. found that patients with first-episode psychosis who had latent iron deficiency had substantially more severe negative symptoms [33]. The symptoms were still significant even after controlling for the length of illness and other confounding factors. Although they were able to prove their hypothesis that abnormal iron metabolism decreased dopaminergic activity and is therefore associated with negative symptoms, they studied cross-sectional data and so the direction of causation cannot be deduced with confidence. Some other limitations of this study were that serum iron and dopamine metabolism in the brain were not backed by the data in the study. Also, there is small direct proof to show that circulating iron levels accurately reflected brain iron levels.

 

Stress-Induced Iron Deficiency

In the previous studies, we have looked at how iron deficiency affects behavioral changes, but one study we came across showed a fascinating dynamic of psychological stress affecting iron availability. Rendina et al. performed a study that examined to see if maternal perceived stress during pregnancy would increase the risk of low neonatal iron at delivery and at one year of age [34]. The study by Monk et al. deduced was that psychological stress could affect many of the exact mechanisms as physiological stressors, including nutritional activities related to iron bioavailability [35]. It is seen that hepcidin-mediated sequestering of iron in maternal tissue could decrease the transfer of iron across the placenta [34]. Also, maternal reaction to stress-inducing activities in pregnancy may initiate inflammatory processes [36] that could interfere with the obligatory four-fold increase in intestinal absorption of iron, thereby impeding placental iron transfer [37,38]. These factors ultimately contributed to iron deficiency in the infants of these mothers; iron levels were checked at delivery and one year of age. While the study by Rendina et al. provided valuable knowledge about maternal stress-causing iron deficiency, it did have some limitations; maternal stress questionnaires were performed after delivery, so the results were retrospective [34]. Another essential thought to consider is that many other studies on animals and humans of gestational stress have deduced even more significant effects of stress during early pregnancy [39-41]. So, to have a better understanding of the effects of maternal stress related to iron deficiency, it would be prudent to perform a more extensive prospective study with repeated reporting of perceived stress throughout all three trimesters of pregnancy, with the sex of the infant taken into account, and also collect repeated health results during the postnatal period.

Iron Deficiency-Induced Social Disengagement

 In one study, East et al. examined how anemic infants pursued and received less stimulation from their caregivers due to iron deficiency [42]. Their findings observed the behavioral ramifications of early nutritional deficiency and its effects on environmental input as contributors to poor results. So essentially, the more infants were socially disengaged and had a lack of effect, the more the parents were disengaged with the children. This resulted in children having lower verbal abilities by age five. The researchers suggested that when medical providers find infants with iron deficiency anemia, they should check for abnormalities in child energy, affect, and social engagement. If there is the presence of some abnormalities, then a dialogue with the parents should be had as to how to engage and stimulate the child properly [43].

Mechanisms of how iron deficiency affects the hippocampus, corpus striatum, and monamines

Iron Deficiency Affecting the Hippocampus

**Figure 1:**
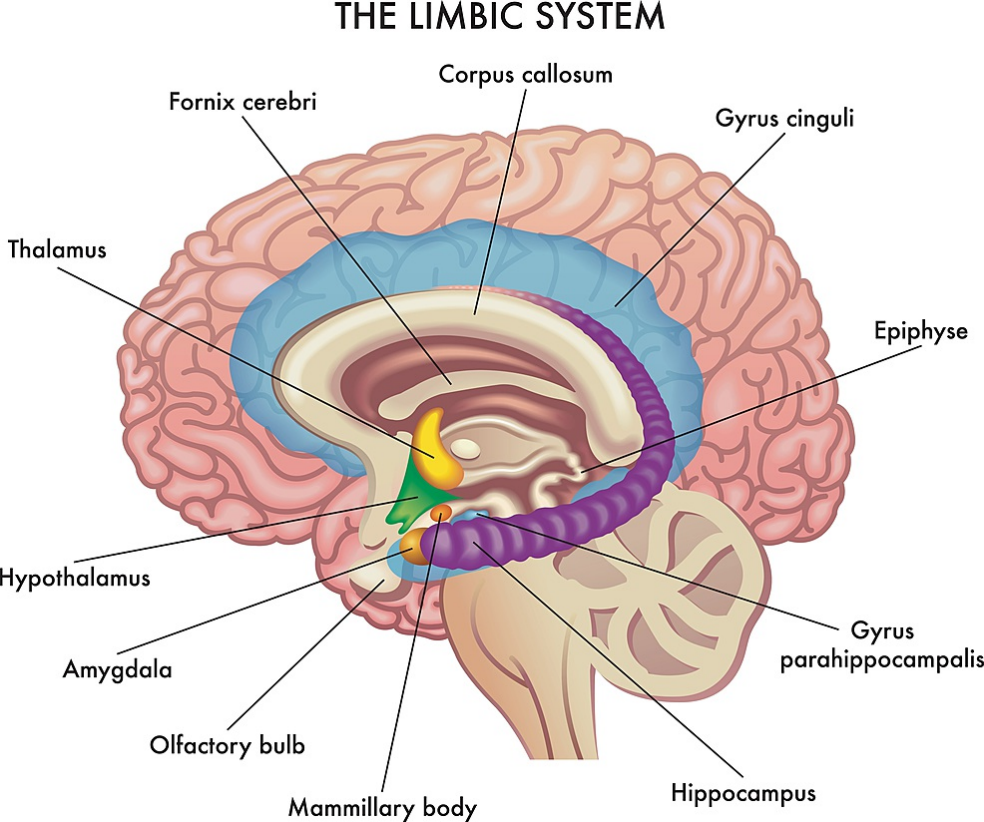
Cross Section of Human Brain Credit: Rob9000. (vector artist).  Medical Illustration Shows The Major Organs of The Limbic System of The Human Brain, With Annotations. [digital image]. Retrieved from www.shutterstock.com [Accessed July 23, 2021]

As we continue our search at mechanisms of how iron deficiency affects behaviors, it has become apparent that the hippocampus is an area of particular interest in relation to iron deficiency and behavioral changes. Animal studies involving rodents have shown that fetal-neonatal iron deficiency has a substantial effect on the hippocampus. The hippocampus is a brain region (Figure 1) that is essential for memory, learning, and other purposes. It was found to be very reactive to lack of Iron during early development [44]. Further research on this effect on the brain by iron deficiency was initially hindered by the difficult task of evaluating brain function in very young infants until some studies started using event-related potentials (ERP) to assess brain function. ERP is a form of neuroimaging that depends on the non-invasive recording of the brain's electrical activity in response to stimulation. In one study, Geng et al. found that infants with normal iron status show electrophysiological confirmation of recognizing their mother's voice but infants with fetal/neonatal iron deficiency did not [45]. In this study, they used electroencephalography to record ERP to deduce auditory recognition memory. It was detected that iron deficiency in the fetal-neonatal period negatively affected recognition memory as quantified by the late slow-wave (LSW). The LSW is an ERP component that is used as a marker of novelty detecting and memory updating. This was an important finding that showed how sensitive to iron deficiency the developing hippocampus is in infants. This was shown initially in animal studies and then confirmed in human studies.

Another study that showed how iron deficiency affects the hippocampus of the developing brain was done by Lien et al. [46]. They found that methylation of DNA plays an essential role in central nervous system development, articulation of normal brain functions, and the etiology of both psychiatric and neurological disorders. DNA methylation is essential for neuronal divergence and maturation in the developing central nervous system, and it also partakes a crucial role in memory and intellect in the adult brain. This study was the first study to ascertain proof of DNA methylation as a possible epigenetic method contributing to hippocampal gene dysregulation in young iron-deficient animals. While there were limitations in this study, including the fact that it was an animal study and they had limited biological replicates because of the high cost of whole-genome bisulfite sequencing, they were able to reconfirm previously studied genes and loci that were changed epigenetically due to iron deficiency and find novel loci essential to neural function that is epigenetically changed by early-life iron deficiency. 

Another mechanism of how iron deficiency can affect behavior is through hippocampal gene expression, as was shown in a study by Burns et al. [47]. They studied *Helicobacter pylori *infection's effect on the hippocampal gene expression. They discovered that *H. pylori* infection has both acute and chronic effects. The acute reaction they saw was increased anxiety behaviors in the open field test at eight weeks post-infection. The chronic reaction was lowering the expression of two hippocampal genes, *Mbp* and *Plp1*. These genes are critical to myelination in the mammalian brain. This indicated that *H. pylori* was an independent factor in changing hippocampal gene expression. It was found the expression of *Mbp* was much lower in brain tissue harvested from *H. pylori*-infected mice compared to the control mice. Protein analysis also showed a similar decrease in Mbp level. This study reinforces that iron deficiency caused by *H. pylori *will ultimately have a profound effect on the behavior and neurologic functions of the studied mice. While this study's main limitation was that it was performed on mice, this was a great study that reinforced data that was studied in the past, and it was the first evidence of *H. pylori*-related inflammation affecting mouse behavioral performances in the open field. 

Iron Deficiency Affecting Corpus Striatum

**Figure 2:**
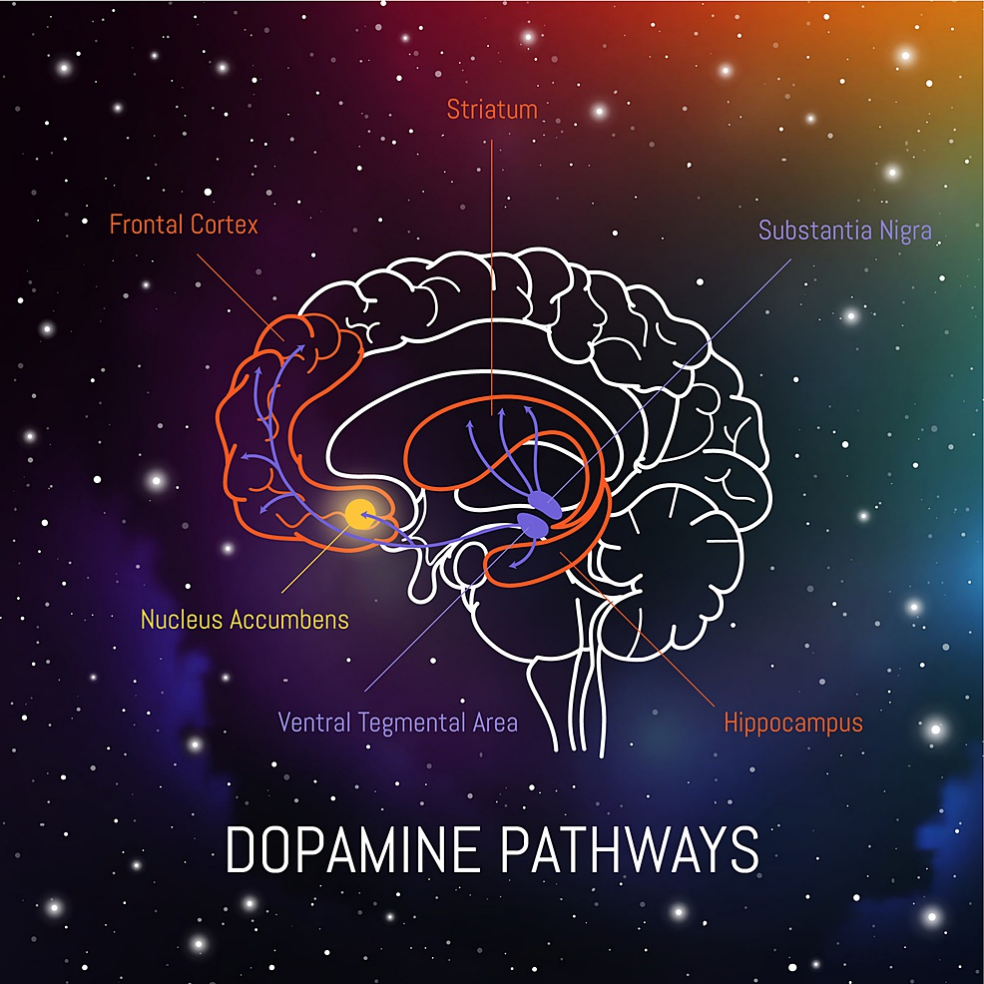
Cross Section of Human Brain: Dopamine Pathways Credit: Veronika. (Illustrator/vector artist). Dopamine Pathways in The Brain [digital image]. Retrieved from www.shutterstock.com [Accessed July 23, 2021]

In addition to iron deficiency affecting the hippocampus in early life, another area that has been found to be affected by early-life iron deficit is the corpus striatum. The corpus striatum and hippocampus, as shown in Figure 2, are two areas of the brain that develop considerably in early life, and both are affected by early iron deficiency in animal studies [48-51]. The corpus striatum dispatches dopamine-rich projects to the prefrontal cortex, and it is involved in controlling executive activities such as planning, inhibitory control, sustained attention, working memory, regulation of emotion, memory storage and retrieval, motivation, and reward. The hippocampus is involved with recognition, recall, and spatial memory. A study performed by Lukowski et al. attempted to show the long-term effect of iron deficiency concerning executive function and recognition memory [52]. They used a neurocognitive battery of tests that included inhibitory control, set-shifting, planning, selective attention, and working memory. They found that even after nearly 20 years of discovering and treating early iron deficiency, specific deficits still persisted. These results are expected with altered functioning of the frontostriatal regions and hippocampus. The study proposes that the effects of neurodevelopment in the first two years of life may determine the long-term higher-level functioning and recognition memory. The study also gives us an interesting insight into how important it is to prevent iron deficiency in pregnancy because even if it is found out in early life and treated, the long-term outcome may still result in a neurocognitive deficit for iron-deficient infants. We also need to continue to pursue other modalities to treat iron deficiency in infancy so we may not have the lasting deficit.

Iron Deficiency Affecting Monoamines

As we continue investigating how iron deficiency affects behaviors, we came across another interesting study by Murat et al. as they looked at the subjective assessment of sleep quality in iron deficiency [53]. Sleep is the period of periodic, physiological, and reversible changes in consciousness and behavior [54]. It is thought that one-third of society has significant sleeping problems. The ratio is higher in the geriatric population and those with psychiatric disorders or learning disabilities [55-58]. Murat et al. hypothesized that sleep quality might decline in iron deficiency anemia because iron plays an important role in the metabolism of monoamines in the brain and those same monoamines play an important role in sleep physiology [53]. Iron deficiency has been known to cause changes in behavioral and developmental aspects by affecting neurotransmitters such as serotonin, noradrenaline, and dopamine. Iron is also related to the myelination and metabolic activity of neurons [59,60]. Peirano et al. delineated that children with iron deficiency anemia displayed longer duration of rapid eye movement (REM) sleep episodes in the first third of sleep and shorter in the last third, more REM sleep episodes in the first third less in the second third, and finally shorter latency to the first REM sleep episode and shorter non-REM stage 2 when compared to the control group [61]. So, they concluded that iron deficiency anemia is related to long-lasting changes in the temporal arrangement of sleep patterns. Peirano et al. surmised that the changes in the neurotransmitter metabolism due to iron deficiency negatively affect sleep [61]. Sleep is also negatively affected by psychological disorders and, possibly, restless leg syndrome. Iron has a complicated effect on dopaminergic functioning. It is a cofactor for tyrosine hydroxylase and is integral to D2 receptor function [7]. Iron deficiency anemia alters dopamine system neurotransmission in specific areas of the brain, among which are those critically involved in sleep regulation. Neuromodulation by the developing dopamine system plays a vital role in sleep control, including the adjustment of REM sleep quality, quantity, and timing [62,63].

Holst et al. studied the sleep-wake regulation in humans and combined pharmacogenetic and neurophysiologic methods to analyze the effects of the 3'-untranslated region (3'-UTR) variable number tandem repeat (VNTR) polymorphism of the gene *SLC6A3* (formerly known as *DAT1*) encoding dopamine transporter (DAT) [64]. The results of their study showed that dopamine transporter contributed to the homeostatic sleep-wake regulation in humans. What Murat et al. ultimately found in their prospective cross-sectional study was that iron deficiency anemia affected sleep quality irrespective of psychological factors [53]. They also concluded that subjective sleep quality was worse in patients with iron deficiency anemia when compared to the control subjects. While this study was interesting and made some valid points, there were a few limitations noted. This study was evaluating sleep quality through self-reporting scales. They also did not find out if treatment of iron deficiency improved sleep quality because of the cross-sectional nature of the study they performed.

## Conclusions

Our review about what behaviors are affected by iron deficiency and how iron deficiency affects behaviors shows the importance of iron in behavioral functions. Iron's effect on behavior begins in utero and continues into the geriatric population. Behavior often seen in infants and children includes wariness and hesitance, lack of positive affect, and diminished social engagement. In adults, anxiety and depression are common. Mechanisms of iron-affected behavior change include changes in functions in the hippocampus, the corpus striatum, and neurotransmitters. From our research, the importance of prenatal iron supplementation in pregnancy is abundantly clear. Even iron replacement in infancy and throughout life may still not correct the damage done to the nervous system in the embryonic state. If we can prevent iron deficiency in utero, it will benefit an individual throughout his life. Studies in the future should focus on enhancing cognitive behavioral therapy and medication that may help resolve ongoing behavior deficits due to iron deficiency.
